# Deep Learning for Type 1 Diabetes Mellitus Diagnosis Using Infrared Quantum Cascade Laser Spectroscopy

**DOI:** 10.3390/ma15092984

**Published:** 2022-04-20

**Authors:** Igor Fufurin, Pavel Berezhanskiy, Igor Golyak, Dmitriy Anfimov, Elizaveta Kareva, Anastasiya Scherbakova, Pavel Demkin, Olga Nebritova, Andrey Morozov

**Affiliations:** 1 Physics Department, Bauman Moscow State Technical University, Moscow 105005, Russia; igorgolyak@yandex.ru (I.G.); dimananfimov97@gmail.com (D.A.); elisabethkareva@gmail.com (E.K.); nastya_schs@mail.ru (A.S.); demkin.pavel1996@yandex.ru (P.D.); o.nebritova@outlook.com (O.N.); amor59@mail.ru (A.M.); 2 Morozov Children’s Clinical Hospital, State Budgetary Healthcare Institution, Moscow Healthcare Pulmonology Department, Moscow 119049, Russia; p.berezhanskiy@mail.ru

**Keywords:** diabetes, breath analysis, deep learning, infrared spectroscopy, quantum cascade laser, biomarker

## Abstract

An estimated 10.5% of the world’s population aged 20–79 years are currently living with diabetes in 2021. An urgent task is to develop a non-invasive express-diagnostics of diabetes with high accuracy. Type 1 diabetes mellitus (T1DM) diagnostic method based on infrared laser spectroscopy of human exhaled breath is described. A quantum cascade laser emitting in a pulsed mode with a peak power of up to 150 mW in the spectral range of 5.3–12.8 μm and Herriot multipass gas cell with an optical path length of 76 m were used. We propose a method for collecting and drying an exhaled human air sample and have measured 1200 infrared exhaled breath spectra from 60 healthy volunteers (the control group) and 60 volunteers with confirmed T1DM (the target group). A 1-D convolutional neural network for the classification of healthy and T1DM volunteers with an accuracy of 99.7%, recall 99.6% and AUC score 99.9% was used. The demonstrated results require clarification on a larger dataset and series of clinical studies and, further, the method can be implemented in routine medical practice.

## 1. Introduction

Non-invasive diagnostics is one of the most important directions for the development of modern medicine. An estimated 537 million adults aged 20–79 years worldwide (10.5% of all adults in this age group) have diabetes, the International Diabetes Federation (IDF) reported in 2021. IDF estimated the number of children (0–19 years) and adolescents with type 1 diabetes to be about 1.2 million in 2021. This number is projected to rise to 643 million by 2030 and 783 million by 2045 [1]. The ability to monitor blood glucose non-invasively by monitoring compounds in breath and emitted through the skin has been demonstrated [2,3]. Recently, the interest has been focused on a compendium of the volatile organic compounds (VOCs) emanating from the human body [4]. VOCs were shown to be isolated from the breath (872 compounds), saliva (359 compounds), blood (154 compounds), milk (256 compounds), skin secretions (532 compounds), urine (279 compounds), and feces (381 compounds) in apparently healthy individuals. Exhaled breath contains many different volatile organic compounds. However, the final list of such substances has not yet been published. A list of compounds that have been observed in breath was published, e.g., by Manolis [5], Philips [6], and Selvaraj [7], including volatile inorganic [8] and organic compounds (VOCs) [9]. For many of these substances it is unknown whether they are produced endogenously, i.e., whether some of them are associated with smoking [10]. Quite a number of volatile compounds may be related to food consumption or medication [11], but some of them can be identified with a possible human disease. Despite the fact that acetone is a biomarker of diabetes mellitus [12], the analysis of acetone alone is insufficient [13]. Others volatile organic compounds such as isoprene and methyl nitrate were associated with diabetes mellitus [14]. The basis of diagnostics is related to disease-specific changes in the concentration of VOCs in exhaled air [15].

The combination of chromatography and mass spectrometry allows rapid identification of substances with high selectivity and sensitivity down to ppt levels [16]. These techniques require accurate calibration of the chromatographic column and manual sampling procedures [17]. Ion mobility spectrometry can be used for breath research [18], but has limitations in separating components in multi-component gas mixtures. Infrared femtosecond lasers can be used for thermal imaging including medical applications [19]. Modern quantum cascade lasers (QCLs) allow to study biomarker molecules with high sensitivity and in the future to create portable devices with low cost of “one measurement” [20,21].

A wide tuning range, emission in the “fingerprint” range, operation at room temperature, and the miniature size of the laser chip make it possible to highlight QC lasers for biomedical applications. Particularly promising is the use of QC lasers in portable devices [22].

In biomedical applications one typically study VOCs at ppb-ppm levels that requires highly sensitive methods. Spectroscopic methods like absorption spectroscopy are limited in sensitivity by the optical path length. Longer path length improves the sensitivity and detection limit. Richard [23] reported the usage of a distributed feedback quantum cascade laser (DFB QCL) at λ∼ 5.26 μm. The NO detection limit of 60 ppt is achieved in a single measurement of 140 ms and an average over 10 s shows sensitivity up to 8.3 ppt. Gorbani et al. [24] used the same system to identify carbon monoxide (CO) in human exhaled breath using a multi-pass gas cell and measured CO at 4.69 μm with a detection limit of 9 ± 5 ppbv and data acquisition time of 0.07 s. McManus [25] reported sensitivity at sub-ppb levels for a narrow band QCL and a 200 m Herriot multipass gas cell.

Aerodyne Research, Inc. (Billerica, MA, USA ) has started commercial production of a compact gas analyzer based on mid-infrared QCL for recording trace amounts of CH${}_{4}$, N${}_{2}$O, NO, NO${}_{2}$, CO, CO${}_{2}$, formaldehyde, formic acid, ethylene, acetylene, ammonia, etc. [26].

In the study [27] using QCL tuning in the 1150–1250 cm${}^{-1}$ range, stable T1DM patients were shown to have concentrations in exhaled breath above the VOC concentration range for healthy individuals. The advantage of using a single biomarker present in high concentrations (e.g., acetone) is obvious, but it alone cannot directly correlate with blood glucose concentrations for all diabetics [3]. Tuzson [28] for a spectral range between 2950 and 2980 cm${}^{-1}$ showed that monitoring acetone in exhaled breath can indeed provide useful information for monitoring of lifestyle interventions. Trefz [29] showed a significant intersection of the values of acetone concentration in exhaled air for diabetic and healthy people, but T1DM patients have significantly higher isopropanol concentrations than their healthy peers.

Another approach is to look at a number of biomarkers and correlate the biomarker pattern (i.e., biomarker combination and their concentrations) [30]. Simultaneous quantification of several gaseous substances enables to observe correlations in their excretion with the exhaled air and, thus, to investigate the interrelationships between various physiological and biochemical processes in the body [31]. E. van Mastrigt [9] for broadband QCL 832–1262.55 cm${}^{-1}$ showed prospects of machine learning methods for diagnosis of asthma and cystic fibrosis for children. Pearson correlation is used to analyze broadband infrared (IR) spectra analysis for remote sensing applications [32,33], but exhaled breath contains a huge number of components and the usage of such methods becomes quite challenging. Machine learning methods are a promising tool for VOC analysis in human breath [34,35]. Kistenev [36] used to apply machine learning for diagnosis of oral lichen planus. Zhu [37] published a current review of deep learning applications for diabetes. It is shown that 610 papers have been published as of 20 October 2020 (the first in 2016). Deep learning methods in medical research are actively developing. Song [38] used neural networks to classify imbalanced oral cancer image data, Zhang [39] used Convolutional Neural Network (CNN) to accurately estimate optical properties of breast tissue in the presence of the chest wall. Deep learning for diabetes diagnosis is a state-of-the-art technique, and it is necessary to conduct extensive experimental and clinical trials to verify the possibility of applying these methods for diabetes diagnosis using QC laser spectroscopy. Moreover, deep learning models are regarded as “black boxes” with a lack of model transparency; therefore, it is necessary to investigate the applicability of deep learning for spectral analysis. Deep learning [40,41] is one of the most effective methods focusing on learning features and building predictive models directly from large-scale datasets [42], and has demostrated success in chemistry, biology, physics, and spectroscopy [43], and metabolomics [44]. CNN is an important branch of deep learning technology inspired by the biological mechanism of visual cognition. For example, Fan et.al. [45] use CNN for Raman spectroscopy applications.

In previous studies [46,47,48] we used machine and deep learning methods to classify and identify VOCs, including multicomponent gas mixtures. The estimated sensitivity of the proposed method was at levels of 10–100 ppb, which makes it possible to diagnose a wide range of diseases using IR laser spectroscopy of exhaled breath.

Our current research is devoted to testing the feasibility of diagnosing T1DM using CNN and IR laser spectroscopy and evaluating the accuracy of the developed method.

In the present paper, an infrared quantum cascade laser and Herriot multi-pass gas cell were used. Infrared spectra from 60 healthy volunteers (the control group) and 60 volunteers with confirmed T1DM (the target group) and used 1-D CNN for volunteer classification were collected. We estimated the accuracy of the diagnosis of type 1 diabetes based on the analysis of exhaled air. We describe in detail the structure and parameters of the neural network and show its capabilities to give researchers an incentive for further work in this area.

## 2. Materials and Methods

### 2.1. Diabetes Fruity Exhaled Breath

T1DM, previously known as juvenile diabetes, is a chronic autoimmune disease characterized by elevated blood glucose levels (hyperglycemia), which are due to the insulin deficiency that results from the loss of β-cells of the islets of Langerhans [49,50]. Type 1 diabetes is a condition in which your immune system destroys insulin-making cells in your pancreas, while type 2 diabetes is a condition in which your body does not respond to insulin the way it should.

The pathogenesis of autoimmune destruction of β-cells is associated with not-fully understood interactions between predisposition genes, autoantigens, and environmental factors. In type 1 diabetes, there is an absolute or relative lack of insulin production. This leads to impaired carbohydrate metabolism as well as metabolic changes such as increased blood glucose levels and intense lipolysis [51]. During lipolysis, fatty acids are quickly mobilized and released from adipose tissue and the synthesis of fatty acids is suppressed in the liver.

Frequently, patients with T1DM are hospitalized with the described symptoms as well as hyperglycemia and sometimes diabetic ketoacidosis (DKA) [52]. DKA most often occurs in patients with T1DM and develops when insulin levels are too low to meet basic metabolic needs. When insulin is deficient, the body receives energy from lipid and amino acid metabolism instead of glucose metabolism. Uncontrolled lipolysis results in increased serum glycerol and free fatty acid levels; the level of alanine also increases due to the catabolism of muscle tissue. Glycerin and alanine serve as substrates for hepatic gluconeogenesis, which is stimulated by excess glucagon accompanying insulin deficiency.

**Figure 1:**
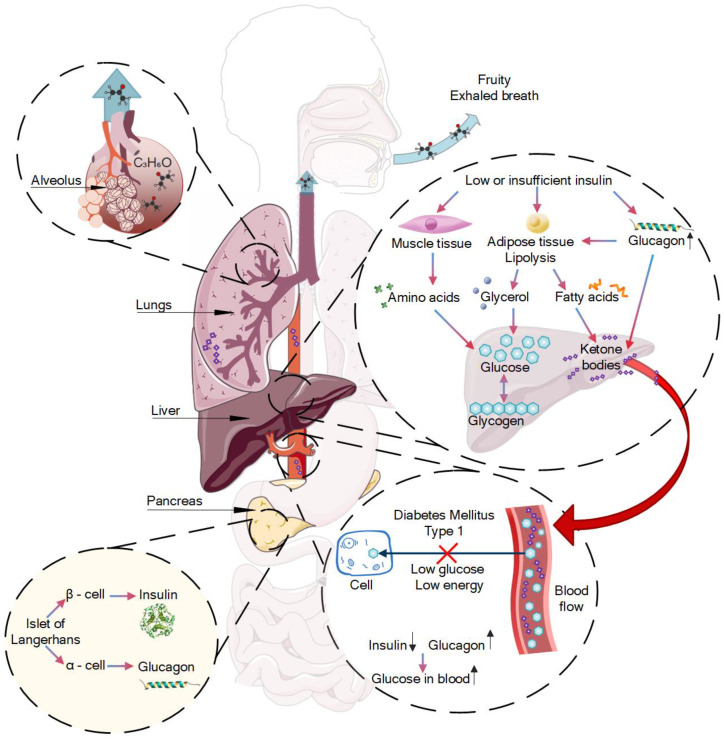
The mechanism of VOC formation in T1DM human breath.

At the same time, glucagon stimulates the conversion of free fatty acids into ketone bodies in the mitochondria. Normally, insulin blocks ketogenesis by inhibiting the transport of free fatty acid derivatives into mitochondria, but ketone bodies are formed in the absence of insulin. The main ones are acetoacetic and beta-hydroxybutyric acids that determine metabolic acidosis. Acetone formed from acetoacetic acid accumulates in the serum and then is slowly excreted through the lungs. The described mechanism causes the specific fruity exhaled breath. Figure 1 shows the mechanism of the appearance of certain VOCs (fruity smell) in T1DM human breath.

### 2.2. Experimental Setup

**Figure 2:**
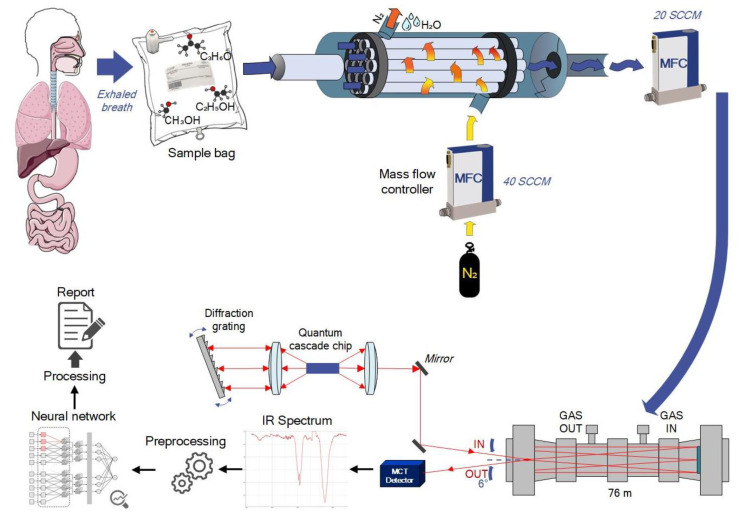
Basic scheme for breath sample analysis method.

T1DM diagnostics is based on the analysis of volunteers’ exhaled breath. Figure 2 shows the basic principle of the developed diagnostic method. An infrared laser spectroscopy to analyze human breath was proposed. The breath sample is collected in a Urine Bag ST 1300102 (Meridian, Moscow, Russia), it is then passed through a Nafion dryer and placed into a Herriot multipass gas cell. IR radiation is emitted by an external cavity quantum cascade laser then it enters to the gas cell and after the required number of reflections is collected at the photodetector. The measured IR spectrum undergoes preprocessing procedures and then comes to the convolutional neural network. A neural network trained on the control and the target groups can classify healthy and T1DM volunteers by their IR breath spectra.

The two mass flow controllers (MFC) type FC-201CV and GE50A (Bronkhorst High-Tech B.V., Bronkhorst, The Netherlands), the pressure controller P-602CV (Bronkhorst High-Tech B.V., Bronkhorst, The Netherlands), and the vacuum pump MVP 015-2 DC (Vacuumbrand GMBH and CO KG, Wertheim, Germany) with pressures up to 3.5 mbar are used. The normal operating pressure is approximately 500 mbar. The exhaled breath must be dehydrated after collection and for this purpose a Nafion gas dryer MD series (Perma Pure LLC, 197 Lakewood, NJ, USA) is used. The pure nitrogen with a flow rate about 40 standard cubic centimetres per minute (sccm) to dry the breath sample and a flow rate of about 20 sccm to place the breath sample into a pre-vacuumed gas cell is used.

**Figure 3:**
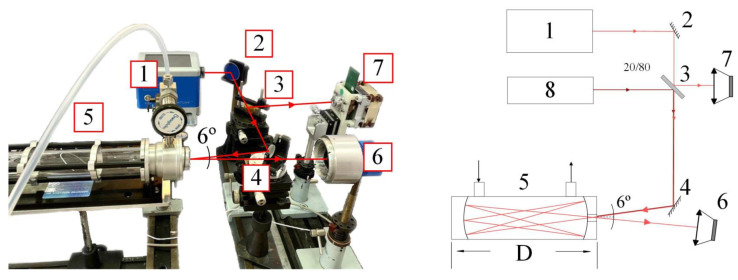
Optical scheme of the experimental setup.

The optical scheme of the experimental setup is shown in Figure 3. The experimental setup is based on the IR quantum cascade laser (Figure 3, pos. 1) and two thermoelectrically cooled photoconductive HgCdTe (MCT-TE) photodetectors (reference photodetector Figure 3, pos. 7, signal photodetector Figure 3, pos. 6, and laser pointer Figure 3, pos. 8). The teference photodetector is used only for adjusting to measure optical path length for the IR beam in the gas cell. Unfortunately, the LaserTune system does not allow to apply an external trigger to use the signal and reference photodetector simultaneously. One signal detector is used for gas analysis and two photodetectors (signal and reference) to determine the optical path by measuring the time delay of the beams traveling to the signal and reference photodetectors. The QC laser (LaserTune, Block Engineering, Southborough, MA, USA) emits in a pulsed mode with a peak power up to 150 mW, a pulse duration of about 50 ns and a repetition rate of about 1 MHz. The photodetector is an MCT-TE photodetector with a detectivity of $D^{*}∼6-8\times 10^{9}$ cm·Hz${}^{1/2}$/W and time resolution of at least 4 ns.

The principle of optical scheme operation is as follows. The laser beam from the QCL (Figure 3, pos. 1) through the mirror (Figure 3, pos. 2) enters the beam splitter (Figure 3 pos. 3), where it is divided into two beams. The first beam falls on the reference photodetector (Figure 3, pos. 7). The second beam through the mirror (Figure 3, pos. 4) enters the gas cell (Figure 3, pos. 5) and, after reflections in the cell, falls on the signal photodetector (Figure 3, pos. 6). The laser pointer (Figure 3, pos. 8) is used when setting up the system to obtain a given pattern of reflections [25], which allows you to obtain the required number of reflections in the gas cell.

### 2.3. Neural Network

In the present paper, the shallow Convolutional Neural Network (CNN) that is a well-known deep learning architecture inspired by the natural visual perception mechanism of living organisms is used.

**Figure 4:**
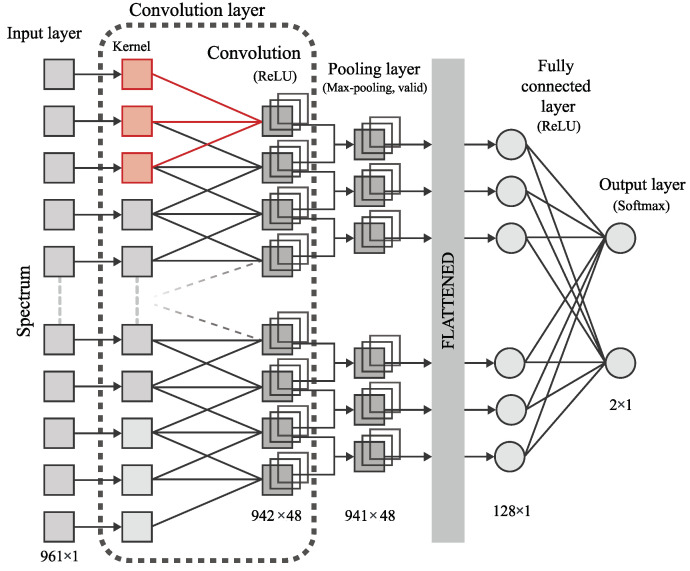
Scheme of the shallow Convolutional Neural Network used in this paper.

Figure 4 shows the shallow convolutional neural network that was created in this work. The proposed CNN model contains an input layer, a single convolutional layer, a max-pooling layer, a fully connected MLP layer (FCL), and the output layer. In this model spectra (one-dimensional raw data arrays) are sent into the input layer. Then these spectra are filtered by the convolutional layer. A one-dimensional kernel is used, because each sample (i.e., spectrum) is represented as a one-dimensional array. The convolution layer uses the ReLU [53] activation function. Then extracted feature arrays are sub-sampled by the max-pooling layer, thus obtaining a reduced optimal feature set. These initial layers represent the feature extraction mechanism. Next comes the flatten layer, where a multidimensional array of features is transformed into a one-dimensional one. After that comes a fully connected multilayer perceptron (MLP) layer with the ReLU activation function and a fully connected output layer with the number of units equal to the number of classes. The use of the softmax activation function on this output layer allows obtaining the class prediction of the network in response to an input sample. The fully connected layers represent the classification mechanism. The stochastic gradient descent (SGD) [54] is used as an updating rule for weights in our neural network. The ‘Glorot’ initialization [55] is chosen for the convolutional kernels and output layer weights because it helps us to keep track of the seed which was used for randomization [56]. The neural network is created using the open-source machine learning library TensorFlow, developed by Google to solve various problems using machine and deep learning methods. API Keras (Google, Mountain View, CA, USA) is also used to build and train models. Therefore the parameters of our model and the range of their values are presented in Table 1.

In order to find the best combination of parameter values, a random grid search cross-validation framework (RGS-CV) [57] is used during the training phase to select the configuration with the highest accuracy. Then the models are refitted using the whole training data and applied to the test data to obtain classification accuracy. Thus, the optimal parameters of our neural network have the following values: kernels = 48, N = 20; s = 1, momentum = 0.9, neurons = 256, lr = $10^{-3}$, epochs = 600.

### 2.4. Groups under the Study

**Figure 5:**
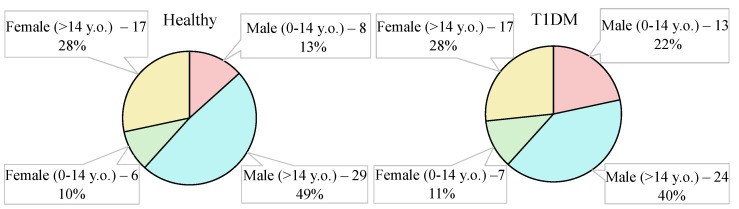
Groups under the study.

Figure 5 shows age and sex charts for the control and target groups. The experimental research was conducted from August to October 2021 on the basis of Bauman Moscow State Technical University (Moscow, Russia) and Morozov Children’s Clinical Hospital State Budgetary Healthcare Institution of Moscow Healthcare Department (Moscow, Russia).

The experimental research was conducted in accordance with the principles of Good Clinical Practices. The protocol of the research was approved by the Ethics Committee of the Morozov Children’s Clinical Hospital State Budgetary Healthcare Institution of Moscow Healthcare Department (Moscow, Russia), Ref. number 174 on 18 January 2022. All participants were informed about details of the research and signed an “informed agreement” for the actions carried out.

Control group: 60 healthy volunteers between the ages of 8 and 21 were examined. All volunteers from the control group had health group 1 based on in-depth preventive examinations. Health group 1 includes persons without any chronic diseases and risk factors for their occurrence. The results of medical examinations in this health group are within the normal range. This category includes citizens with the most favorable level of health. Based on the results of medical examination, preventive consultations and other medical and recreational activities are carried out for persons in this category, with the main purpose of promoting a healthy lifestyle and observing sanitary and hygienic standards.

The target group: 60 patients aged 6 to 17 years were examined. All volunteers had an average degree of severity of the disease, four volunteers had acute ketoacidosis, the rest had decompensation stage without ketoacidosis. The average glucose level at admission is 13.05 mmol/L (from 7.3 to 38 mmol/L). Diabetes experience: Average 7.7 years (from 1 year to 15 years).

### 2.5. Sampling Protocol

Exhaled breath samples were taken on an empty stomach without morning oral hygiene procedures at a room temperature of 20–22 °C. A disposable Urine Bag ST type 1300102 (Meridian, Moscow, Russia) was used for breath sampling. The volunteer exhaled the volume of their usual breath into the bag without taking a deep breath beforehand. Volunteers were asked to avoid inhaling through the nose while exhaling through the mouth due to the “lack” of air reflex.

Volunteers exhaled as much as they could into two-liter valve bags. Since the volume of the gas cuvette is 0.5 liters and the operating pressure is about 500 mbar, the volume of one exhalation is sufficient for sample analysis.

The preservation of the sample was checked in the urine bag and it was experimentally established that the correlation coefficient of the sample of the infrared spectrum of the volunteer’s air sample immediately after taking the sample and after 8 h of storage in the sample bag is 0.97. This allowed us to transport the breath sample from a medical facility to the laboratory.

## 3. Results

### 3.1. Sensitivity of Experimental Setup

The diagnosis of T1DM by human breath analysis is based on detecting the presence of certain biomarker molecules (or its patterns), as well as on the excess of their concentrations of a certain threshold. Thus, the developed experimental setup must have a sensitivity (minimally detectable concentrations) no worse than the values of the certain threshold corresponding to certain VOC and diseases. The relationship between the components of exhaled air and human health pathologies is well known [3]. The average acetone concentration in healthy breath varies from 293 to 870 ppb and ethanol from 27 to 153 ppb [58]. Average acetone concentration may exceed 1800 ppb for diabetic patients [59]. P. Trefz et al [29] reported that T1DM patients exhaled significantly higher amounts of ethanol, isopropanol, dimethyl sulfide, isoprene, and pentanal compared with healthy controls (171, 1223, 19.6, 112, and 13.5 parts per billion by volume (ppbv) vs. 82.4, 784, 11.3, 49.6, and 5.30 ppbV). M. Simic [60] reported that endogenous ethanol correlates with increased glucose blood levels and can alert about T1DM. Acetone and ethanol as major biomarkers for T1DM are examined.

**Figure 6:**
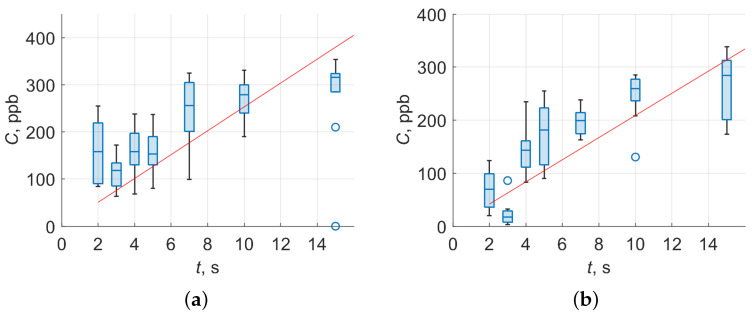
Ethanol (**a**) and acetone (**b**) minimum detectable concentrations for test gas mixtures.

A standard gas mixture with pure nitrogen with a concentration of 1000 ppm for acetone and ethanol is used. First, the gas cell is pumped to a pressure of 1 mbar. Then the gas mixture is fed from the cylinder at a given rate. The substance can be identified in the described experimental setup if the correlation coefficient of the experimental and base spectrum (registered at a high concentration of about 50–100 ppm) is at least 0.5 (corresponding to a time value of 2 s on Figure 6). The value of the minimum detectable concentration is determined by calculating the flow rate of the gas mixture (red straight line in Figure 6) and according to the Beer–Lambert law (box plot in Figure 6). To calculate the concentration according to the Beer–Lambert law, the absorption cross-sections for some VOCs at a given wavelength (Table 2) is experimentally determined. The systematic error and measurement techniques cause different slopes of experimental results in Figure 6.

The minimum detectable concentrations for ethanol and acetone were experimentally obtained at levels 51 and 42 ppb using the gas mixture flow rate calculations (red line on Figure 6) and 157 and 67 ppb as median values for box plot Figure 6, with rms values equal to 63 and 41 ppb for ethanol and acetone, respectively. The obtained results allowed us to assert that the developed experimental setup makes it possible to detect typical T1DM molecule biomarkers at the required concentration.

### 3.2. Classification of Volunteers by Infrared Breath Spectra

**Figure 7:**
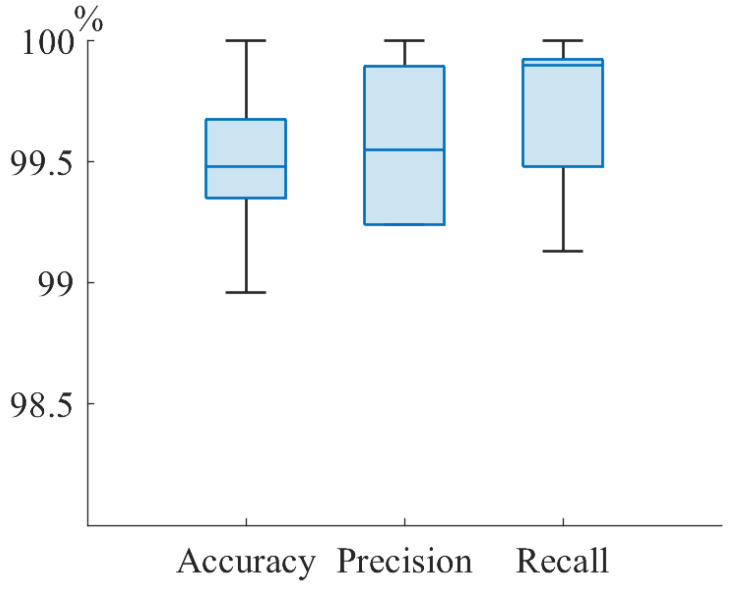
CNN cross-validation results on the training dataset.

A balanced dataset consisting of 60 healthy volunteers and 60 T1DM volunteers aged from 6 to 21 years was used. Breath samples of T1DM volunteers with type I diabetes were taken in Morozov Children’s Clinical Hospital State Budgetary Healthcare Institution of the Moscow Healthcare Department. Ten measurements were carried out with each volunteer. Each measurement represents the spectrum of the exhaled air. A total of 1200 spectra were obtained, including 46 girls and 74 boys (34 children under 14 years old and 86 children over 14 years old). Of the total number, 60% was taken for training, 20% of the total number was taken for validation, and 20% of the total number of spectra was taken for testing. Accuracy, which is an estimate of the probability that an arbitrary object is classified correctly, was chosen as a metric for determining the quality of classification by a neural network. To achieve the highest accuracy of the neural network, calculations were conducted with the next optimum parameters: kernels = 48, N = 20, s = 1, momentum = 0.9, neurons = 256, lr = $10^{-3}$, epochs = 600. To evaluate the effectiveness of the obtained neural network parameters, cross-validation was performed on the entire training data. The results of CNN training on Figure 7 are shown. The graph from Figure 7 shows that the median accuracy was at least 99.5% on training dataset. After that, the trained neural network on the remaining 20% test sample consisting of 24 people (240 spectra) was applied. The results of T1DM and healthy volunteer classification by infrared breath spectra are shown in the Table 3. The Table 3 shows the probability that an arbitrarily taken T1DM volunteer is classified correctly (sensitivity, recall) is no less than 99%. Moreover, a probability that an arbitrarily taken volunteer is classified correctly (accuracy) of at least 99% was achieved.

Area under the curve (AUC) score for convolutional neural network classification of healthy and T1DM volunteers for all sex and age groups achieved no less than 99.9%. This result shows that a randomly selected object can be positively classified with a high probability based on its IR spectrum. In contrast, in the spectra of healthy and T1DM volunteers, the neural network finds stable features necessary for classification. IR breath spectra using a neural network and selected optimal parameters for high accuracy were analyzed. As a result, the highest accuracy in the analysis of all volunteers was achieved, dividing them into healthy and T1DM volunteers (99.6%). The use of training and cross-validation on the entire data volume was shown. The expected reduction of the test group within one gender slice should increase the classification accuracy. However, the experiments showed that the classification accuracy for the entire dataset appears to be the highest compared to the slices. This can be explained by the group size, which directly affects the classification accuracy. It is possible to use data augmentation [61] to increase the dataset, which can improve the accuracy of the neural network.

Advanced exhaled air diagnostic methods reveal a large number of VOCs. Changes in their levels are frequently linked to specific diseases or metabolic disorders in general. The determination of VOCs to search for prognostic markers for the development of metabolic disorders, particularly diabetes mellitus, is promising. The use of such predictors in screening large population groups and developing preventive measures on this basis is a significant social as well as biomedical issue, particularly when it comes to children’s health. Acetone is one of the potentially volatile compounds linked to metabolic abnormalities. Variations in its content in exhaled air or urine fairly accurately reflect changes in lipid metabolism, particularly lipid beta-oxidation. Type 1 diabetes mellitus occurs when the pancreatic β-cells that produce insulin are destroyed by the immune system, necessitating lifelong insulin therapy. Patients use home glucose meters to determine if they need to administer insulin, and the ISO 15197 standard for available glucose meters allows a margin of error of ±20% error. Therefore, it is very important to develop other ways to control diabetes.

As a result of the conducted research, it is clear that the use of infrared laser spectroscopy is promising for the development of express methods for analyzing diabetes mellitus biomarkers in large-scale surveys in order to implement appropriate preventive and therapeutic interventions. However, more precise identification of the corresponding gas-metabolic profiles in diabetic patients representing systemic metabolic rearrangements under normal and pathological conditions is needed, because light hydrocarbons are intermediate or by-products of numerous metabolic cycles [62]. Acetone, for example, is formed as a result of the involvement of fatty acids in the energy metabolism of diabetes mellitus [63]. With starvation, prolonged intensive physical work [51], and changes in the enteric environment [64], more acetone can be formed in exhaled air [65]. The properties of light hydrocarbons in exhaled air can be used to predict individual metabolic features, including those associated with risk factors for metabolic disorders [6]. The assessment of acetone in exhaled air in conjunction with the clinical picture can be a reliable marker of liver damage and necrosis, representing the severity of oxidative stress along with the content of glycated hemoglobin in blood and products of lipid peroxidation. There are marked dysmetabolic disorders in diabetes mellitus patients’ connective tissue, in the endothelium, where active metabolites produced by oxidative stress potentiate the formation of volatile organic compounds such as ethane and pentane, the assessment of which will also be relevant in exhaled air, as it will help determine dysmetabolic changes in express mode without taking biochemical blood tests. It is possible to create a gas metabolic profile of diabetic-diabetes mellitus patients. The analysis of the whole spectrum of exhaled breath as a pattern of components as well as various biomarkers for human health check is promising.

We understand that the target group contained children in the acute stage of diabetes. At this stage, we have tested the method and evaluated its accuracy. However, for early diagnosis, it is necessary to create a target group with blood glucose values close to the control group.

## 4. Conclusions

Infrared laser spectroscopy to analyze human exhaled air was used. The experimental setup consisted of a quantum cascade laser emitting in a pulsed mode with a peak power up to 150 mW in the spectral range of 5.3–12.8 μm and a Herriot multipass gas cell with an optical path length 76 m. The control group included 60 healthy volunteers aged from 8 to 21 years; the target group included 60 volunteers with confirmed T1DM aged from 6 to 17 years. A method for collecting and drying an exhaled human air sample and collecting 1200 infrared breath spectra (10 spectra for each of 120 individuals) was proposed. The 1-D convolutional neural network to classify healthy and T1DM volunteers using IR breath spectra was used. The whole IR breath spectra of each volunteer for analysis was used. The optimal parameters of the neural network were obtained: kernels = 48, N = 20, s = 1, momentum = 0.9, neurons = 256, lr = 10${}^{-3}$, epochs = 600. With an optimally tuned neural network, we achieved the probability that an arbitrarily taken T1DM volunteer is classified correctly (recall) is no less than 99%. Moreover, the achieved probability that an arbitrarily taken volunteer is classified correctly (accuracy) is at least 99%. The area under the curve score for convolutional neural network classification of healthy and T1DM volunteers for all sex and age groups achieved no less than 99.9%. The obtained data require clarification on a larger sample as well as investigation of the possibilities of diagnosing other diseases. The most urgent task is to develop criteria for early rapid diagnosis of patients in prediabetic condition.

We hope that the proposed experimental setup and neural network can be used to create devices that will be used in routine medical research as a doctor’s decision-making assistance system.

## Figures and Tables

**Table 1:** Ranges of values for the convolutional neural network model.

Parameter	Value	Value Range
Number of kernels of the convolutional layer	kernels	{24,48}
Size of kernels of the convolutional layer	N	[10,20]
Stride for the convolution and max-pooling	s	[1,2];
Momentum in the SGD updating rule	momentum	[0.1,0.9]
Number of neurons in FCL	neurons	[128,256]
Learning rate	lr	$\left[10^{-3},10^{-4}\right]$
Number of epochs	epoches	[100,600]

**Table 2:** The molecular cross-section for some VOCs.

No	Substance	Wavenumber, cm${}^{-1}$	Cross-Section, 10${}^{-19}$ cm${}^{2}$
1	Ammonia	930	4.28
2	Acetone	1217	3.37
3	Methanol	1033	7.16
4	Ethanol	1065	2.58

**Table 3:** Results of T1DM and healthy volunteer classification by infrared breath spectra.

Group	Accuracy	Precision	Recall
All	99.7	99.5	99.6
Male	99.5	98.6	100
Female	99.7	99.9	99.8
Minors (less 14 y.o.)	99.5	99.6	99.5
Adults (more 14 y.o.)	98.9	99.3	99.3

## Data Availability

The data presented in this study are available on request from the corresponding author.

## Abbreviations

The following abbreviations are used in this manuscript:

AUC	Area under the curve
CNN	Convolutional neural network
DFB	Distributed feedback
DKA	Diabetic ketoacidosis
IDF	International Diabetes Federation
IR	Infrared
FCL	Fully connected layer
MIR	Mid-infrared
MCT-TE	Mercury Cadmium Telluride thermoelectrically cooled
MFC	Mass flow controller
MLP	Multilayer perceptron
QCL	Quantum cascade laser
ppb	Parts per billion
ppbv	parts per billion by volume
ppm	Parts per million
RGS-CV	Random grid search cross-validation framework
sccm	standard cubic centimetres per minute
SGD	Stochastic gradient descent
T1DM	Type 1 diabetes mellitus
VOC	Volatile organic compound
y.o.	Years old
